# The burden of disease from air pollution in Israel: How do we use burden estimates to advance public health?

**DOI:** 10.1186/s13584-016-0120-5

**Published:** 2016-12-16

**Authors:** Jonathan M. Samet

**Affiliations:** Department of Preventive Medicine, Keck School of Medicine of USC, USC Institute for Global Health, University of Southern California, Soto Street Building, Suite 330, 2001 N Soto Street, MC 9239, Los Angeles, CA 90089-9239 USA

## Abstract

In an article recently published in the IJHPR, Ginsberg and colleagues from Israel’s Public Health Services estimate the disease burden from airborne particulate matter in Israel. Using national data on the concentration of PM_2.5_ (particulate matter less than 2.5 μm in aerodynamic diameter) and risk estimates from meta-analyses, they calculate that about 2000 deaths (4.7% of total deaths) are attributable to air pollution. Although inherently subject to uncertainty, such estimates are useful for motivating public health protection and gauging the stringency of any needed regulations. However, Israel does not yet have an evidence-based process for air quality regulation comparable to that of the United States, which has evolved over the 45 years since passage of the Clean Air Act. In fact, Israel has only recently promulgated a national standard for airborne particulate matter and quantitative risk assessment has not been an element of regulatory decision-making. The report by Ginsberg and colleagues represents a useful beginning and should initiate discussion of the role of burden estimation and risk assessment more broadly in regulations intended to advance environmental health in Israel.

## Background

In 1953, Morton Levin published a landmark paper that described a measure that is widely known as the population attributable risk, a parameter that gives an estimate of the burden of disease attributable to a particular factor in a population [1]. At the time, the emerging literature on smoking and lung cancer had convinced Levin that the relationship was causal. He reasoned that if a factor caused a disease than an immediate question was its overall importance as a cause, leading him to the formula that is still used for this purpose. Application of Levin’s formula requires specification of the exposure and the associated risk for disease of that exposure and also of a comparison or counterfactual exposure, reflecting a theoretical minimum exposure that might be achieved. In the example of cigarette smoking and lung cancer, the values for these elements of Levin’s formula are readily specified: there are many estimates of the relative risk of lung cancer for smokers compared with nonsmokers and the counterfactual comparison is obvious—a smoking prevalence of zero. Thus, current estimates of the fraction of lung cancer attributable to smoking in high-income countries are around 85% percent, given ever-smoking prevalence figures of about 30–40% and relative risk estimates of 20 and about 10 for current and former smokers, respectively, compared with never smokers [2].

Many decades later and in an article which was recently published in the IJHPR, Ginsberg and colleagues [3] from Israel’s Public Health Services use the same conceptual approach to estimate the disease burden from airborne particulate matter in Israel. For ambient or outdoor air pollution, the conceptual basis of the calculation is similar to its application to smoking, but there is greater uncertainty around the values for exposure and risk compared with the case of cigarette smoking. For exposure, Ginsberg et al. report that the 2015 mean concentration of PM_2.5_ (particulate matter less than 2.5 μm in aerodynamic diameter) was 21.6 μg/m^3^; this figure is applied to all residents of Israel, ignoring the range of variation of actual exposures to particulate matter across the population. Another key matter is the selection of a counterfactual value for Israel; on this choice, the authors default to the World Health Organization (WHO) methodology cited, which incorporates comparison values ranging from 5.8 to 8.8 μg/m^3^ [4]. The choice of the counterfactual value is critical in interpreting the findings: is it achievable? or, does it represent a goal, such that the burden estimated exceeds what might be achieved through practicable measures for exposure reduction? For the United States, as the Environmental Protection Agency has examined potential revision of the National Ambient Air Quality Standard (NAAQS) for ozone, there has been substantial controversy concerning the appropriate counterfactual background concentration for risk assessment, given the natural production of ozone and the long-range transport of ozone into the United States [5]. For PM_2.5_, justification of a particular counterfactual value for Israel is problematic, given the country’s geography and the extent of pollution transport into the country. Thus, through air quality management within the country, Israel may not be able to reach the WHO counterfactual values through national actions alone.

The other element of the calculation is the selection of risk values for the adverse effects of air pollution; these are in the form of percentage change in risk for disease in relation to PM_2.5_ concentration. One challenge faced by Ginsberg and colleagues was the selection of the risk coefficients, which ideally would come from locally-based investigations. The authors addressed this challenge, lacking locally generated estimates, by turning to various systematic reviews and meta-analyses related to air pollution and the associated risks for the specific diseases for which burden was to be calculated. Their selection methods are described, although not with the detail needed to replicate fully their approach. An important issue is the generalizability of the findings of the risk estimates, which come from studies of populations outside of Israel, to the population of Israel. This generalization of the risk estimates is further complicated by the substantial diversity of the Israeli population. Meta-analyses of epidemiological studies of the health effects of air pollution generally show variation in risks among studies [6]. Thus, the selection of particular risk estimates for burden estimation is an inevitable and not readily quantified source of uncertainty.

The approach taken by Ginsberg and colleagues deviates from that used by the World Health Organization and the Global Burden of Disease program (http://www.healthdata.org/gbd). Burden estimates made by these entities for the major disease outcomes are based on so-called integrated exposure-response functions (IER) developed for ALRI (acute lower respiratory infections), lung cancer, COPD (chronic obstructive pulmonary disease), and cardiovascular disease. These IER functions are based on a combined analysis of multiple relevant data sets; for example, bringing together data on active and passive smoking and air pollution for lung cancer, COPD and cardiovascular disease [7]. In estimating global disease burden, the IER risk relationships are applied uniformly across all countries, potentially not appropriately capturing risk for some countries.

### How are burden estimates used?

The initial use of attributable risk calculation for cigarette smoking established one paradigm for policy application: use of burden estimates as an imperative for action. In the United States, the burden of premature death from active and passive smoking has been closely tracked and served as a reminder of the need for sustained and aggressive tobacco control. Burden estimation has now become a global tool, used by the WHO and the Global Burden of Disease (GBD) initiative at the Institute for Health Metrics and Evaluation in the United States [4, 8]. The GBD estimates have provided an understanding of the comparative importance of different contributors to disease burden and changes in these contributions over time. In the GBD 2013 estimates, ambient air pollution and household air pollution, have surprising prominence; ambient air pollution accounts for 4.2 million attributable deaths or 12% of total deaths of disease burden while the count for household air pollution is 2.9 million deaths or 5% of the total. There are an additional 250,000 deaths attributable to ozone. For Israel, the GBD estimate of attributable deaths for 2013 to ozone and particulate matter is 2250 or about 5% of total deaths [8].

For decades, quantitative risk assessment has been used for decision-making concerning environmental hazards in the United States and elsewhere. As codified in the “Red Book”, a 1983 report from the US National Academy of Science, quantitative risk assessment has four components: hazard identification (does an agent pose a risk?), dose–response (how does risk vary with exposure or dose?), exposure assessment (what is the distribution of exposure to the agent?), and risk characterization (what is the risk posed to the population) [9]. The latter component is conceptually comparable to burden estimation as implemented by Ginsberg and colleagues. As described below, quantitative risk assessment is incorporated into the process used in the United States for NAAQS revision.

Why have Ginsberg and colleagues, who work for a national public health agency made estimates of disease burden associated with ambient air pollution? The estimates are offered with the rationale that “Measuring the burden of disease from air pollution is important not just for advocacy but also as a first step towards carrying out a full cost-effectiveness analysis in order to prioritise technological interventions that are available to reduce air pollution…” The results are used to justify a conclusion that the burden from air pollution “…cries out for the establishment of an inter-ministerial plan to identify and implement those intervention strategies that are cost-effective…” This call for strategies for air quality management in Israel assumes that the burden is too high and can be reduced. Given that the models used for burden estimation are linear without threshold, air pollution will necessarily contribute to disease burden; from the policy perspective, one complicated matter is what level of burden is to be avoided through air quality management and what residual burden that cannot be controlled is acceptable? This is a societal judgment that is generally made through precedent, still lacking in Israel. The total calculated by Ginsberg et al. of about 2000 deaths constitutes 4.7% of total deaths in Israel in 2015, comparable to the GBD estimate. They have judged that figure to warrant action.

Experience from the United States documents one way to use the burden estimates for air quality regulation. The calculations of avoidable burden have now been incorporated into the process for revising the NAAQS. In a multi-step process of evidence translation, a risk and exposure assessment is carried out to estimate the extent to which potential changes in the NAAQS would reduce burden and also the burden of disease remaining if the revised NAAQS were achieved [5]. In revising the NAAQS, the Administrator of the Environmental Protection Agency considers the public health benefits and the residual disease burden for alternative values of the standard [10]. Costs do not figure directly in the Administrator’s decision, raising the question as to how the results of a “full cost-effectiveness analysis” would be used in decision-making in Israel. Doing so would require guidance on the parameters of cost-effectiveness suitable for the context of decision-making in Israel.

## Conclusions

Israel does not yet have an evidence-based process for air quality regulation comparable to that of the United States, which has evolved over the 45 years since passage of the Clean Air Act. In fact, Israel has only recently promulgated a national standard for airborne particulate matter and quantitative risk assessment has not been an element of regulatory decision-making [11]. The report by Ginsberg and colleagues represents a useful beginning and should initiate a discussion of the role of burden estimation and risk assessment more broadly in regulations intended to advance environmental health in Israel. The findings point to the need to set out on an agenda for air quality management, as called for by Ginsberg et al.
